# Vascular Senescence: A Potential Bridge Between Physiological Aging and Neurogenic Decline

**DOI:** 10.3389/fnins.2021.666881

**Published:** 2021-04-20

**Authors:** Sara Rojas-Vázquez, Laura Blasco-Chamarro, Irene López-Fabuel, Ramón Martínez-Máñez, Isabel Fariñas

**Affiliations:** ^1^Instituto Interuniversitario de Investigación de Reconocimiento Molecular y Desarrollo Tecnológico (IDM), Universitat Politècnica de València-Universitat de València, Valencia, Spain; ^2^Centro de Investigación Biomédica en Red de Bioingeniería, Biomateriales y Nanomedicina (CIBER-BBN), Valencia, Spain; ^3^Departamento de Biología Celular, Biología Funcional y Antropología Física, Universitat de València, Valencia, Spain; ^4^Instituto de Biotecnología y Biomedicina (BioTecMed), Universitat de València, Valencia, Spain; ^5^Centro de Investigación Biomédica en Red de Enfermedades Neurodegenerativas (CIBERNED), Madrid, Spain; ^6^Unidad Mixta UPV-CIPF de Investigación en Mecanismos de Enfermedades y Nanomedicina, Universitat Politècnica de València, Centro de Investigación Príncipe Felipe, Valencia, Spain; ^7^Unidad Mixta de Investigación en Nanomedicina y Sensores, Universitat Politècnica de València, IIS La Fe, Valencia, Spain

**Keywords:** adult neural stem cell, neurogenic niche, endothelial cell senescence, senescence-associated secretory phenotype, parabiosis

## Abstract

The adult mammalian brain contains distinct neurogenic niches harboring populations of neural stem cells (NSCs) with the capacity to sustain the generation of specific subtypes of neurons during the lifetime. However, their ability to produce new progeny declines with age. The microenvironment of these specialized niches provides multiple cellular and molecular signals that condition NSC behavior and potential. Among the different niche components, vasculature has gained increasing interest over the years due to its undeniable role in NSC regulation and its therapeutic potential for neurogenesis enhancement. NSCs are uniquely positioned to receive both locally secreted factors and adhesion-mediated signals derived from vascular elements. Furthermore, studies of parabiosis indicate that NSCs are also exposed to blood-borne factors, sensing and responding to the systemic circulation. Both structural and functional alterations occur in vasculature with age at the cellular level that can affect the proper extrinsic regulation of NSCs. Additionally, blood exchange experiments in heterochronic parabionts have revealed that age-associated changes in blood composition also contribute to adult neurogenesis impairment in the elderly. Although the mechanisms of vascular- or blood-derived signaling in aging are still not fully understood, a general feature of organismal aging is the accumulation of senescent cells, which act as sources of inflammatory and other detrimental signals that can negatively impact on neighboring cells. This review focuses on the interactions between vascular senescence, circulating pro-senescence factors and the decrease in NSC potential during aging. Understanding the mechanisms of NSC dynamics in the aging brain could lead to new therapeutic approaches, potentially include senolysis, to target age-dependent brain decline.

## Introduction

**FIGURE 1:**
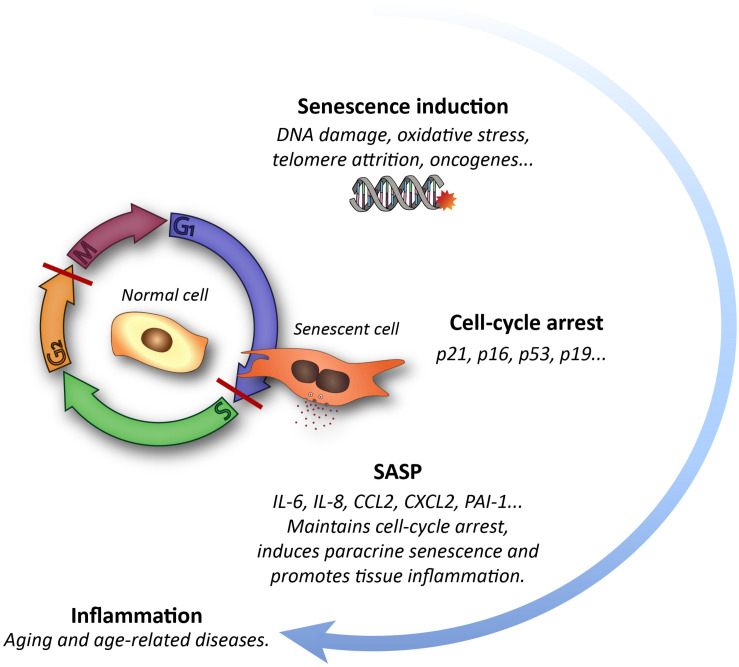
Schematic representation of the cellular senescence process and its impact on tissues.

Aging is envisioned as a progressive decline in the function of multiple tissues and organs that leads to disruption of homeostasis and increased frailty (López-Otín et al., 2013). One of the hallmarks of aging is a rise in the frequency of senescent cells in most organs (Childs et al., 2017). Cells undergo senescence in response to several stressors, including telomere attrition and other forms of DNA damage, oncogene activation, epigenetic remodeling, hypoxia, oxidative stress or mitochondrial dysfunction (Hernández-Segura et al., 2018). Cell senescence is an irreversible arrest of the cell cycle that is accompanied by an increase in heterochromatin and in foci of DNA damage-responsive proteins, or DNA scars, and increased levels of cyclin-dependent kinase inhibitor (CKI) proteins p16^*INK*4*a*^ and/or p21^*CIP*1^. Senescent cells also exhibit larger cell size and an augmented lysosomal compartment, with increased levels of the lysosomal enzyme β-galactosidase (β-gal) (Kurz et al., 2000; Childs et al., 2017; Herranz and Gil, 2018). Although these cells do not proliferate, they persist in the tissues and exhibit an intense secretory activity named senescence-associated secretory phenotype (SASP). Their secretome is composed of a complex mixture of molecules, including pro-inflammatory cytokines and chemokines as well as proteases, which are released to the extracellular space creating a pro-inflammatory environment that can negatively influence neighboring cells and overall tissue biology (Table 1 and Figure 1; Van Deursen, 2014; Muñoz-Espín and Serrano, 2014; Childs et al., 2017). The accumulation of senescent cells with advancing age in the tissues reflects the decline in cell repair mechanisms and in the capacity of the immune system to clear both damaged and senescent cells (Childs et al., 2017). This is in part due to the fact that these cells develop pro-survival pathways to avoid the recognition that lead to immune clearance (Yosef et al., 2016). An increased burden of senescent cells results in functional cell loss and a wide variety of non-autonomous disturbances that correlate with a deterioration of tissue renewal and performance, favoring organ dysfunction. Therefore, senescence has emerged in the recent years as a driver of aging and age-related diseases (Childs et al., 2017).

**TABLE 1:** Compilation of the main phenotypic features of senescent cells and consequences of their age-related accumulation.

Cellular phenotypic changes	Cell senescence	Senescence-associated markers	Consequences in aging	References
Structural disturbances	Large flattened morphology.	*In vitro* cultures: large size, flattened shape, various processes, multi-nuclei (sometimes), numerous citoplasmatic vacuoles. These features may change depending on the cell type and the inductor agent.	Changes in the shape and morphology of senescent cells *in vivo* are not yet well documented. However, if these cells undergo structural alterations *in vivo*, their function is also compromised. For example, the ECs’ tight junctions could be altered by changes in cell structure, which could impair the integrity of the BBB.	Muñoz-Espín and Serrano, 2014; Yamazaki et al., 2016; Hernández-Segura et al., 2018
Cell-cycle arrest	Irreversible cell-cycle arrest impedes senescent cell proliferation.	p21, p16, p19, p53, pRB.	CDKi induce cell growth arrest permanently in response to different stressors or DNA damage. With age, the incidence of non-pro-liferative senescent cells increases and stem cells undergo exhaustion in the attempt to regenerate tissue, which can lead to tissue fibrosis.	Childs et al., 2017; Herranz and Gil, 2018
Nuclear changes	Telomere attrition and DNA damage response: chromatine modifications and nuclear lamina disturbances.	Telomere-associated foci (TAF), γH2AX, 53BP1, loss of lamin B1.	The accumulation of DNA alterations triggers a DNA damage cell response that induces cell-cycle arrest and promotes changes at the transcriptional level defining the senescent phenotype.	Van Deursen, 2014; Hernández-Segura et al., 2018
Altered metabolism	- Increased lysosomal cell compartment. - Senescence-associated secretory phenotype (SASP): cytokines, chemokines, proteases, growth factors, insoluble proteins (ECM). - Altered autophagy.	- Senescence-associated β-galactosidase activity (SA-p-gal). - SASP mediators: p38MAPK, mTOR, NFκβ, CGAS/STING. - SASP secreted factors: IL-6, IL-1a, IL-1b, IL-8, IL-11, CCL2, CCL5, CXCL1, CXCL2, CXCL5, TNF-α, TGF-β, MCP-1, PAI-1. - Decreased autophagy markers: Atg5, LC3I, LC3II.	- Senescent cells overexpress the lysosomal enzyme β-gal. The high lysosomal content of senescent cells may also contribute to their potent SASP. - SASP promotes inflammation and oxidative stress, mediates transcriptional changes in adjacent cells with deleterious effects on tissue function and induces cell senescence via paracrine manner. - Senescence is associated with decreased cellular autophagy, for example, in immune cells have been reported an impaired autophagy with aging.	Kurz et al., 2000; Coppé et al., 2010; Zhang et al., 2017

Populations of resident stem cells (SCs) in adult tissues are characterized by their ability to self-renew while producing specialized cell progeny, thereby sustaining life-long physiological cell turnover and recovery from injury. Despite their lasting activity, aging also has a negative impact on SC maintenance and potential to produce descendants (Schultz and Sinclair, 2016). Because SCs are at the top of the cell lineage hierarchy of mature cell production in many tissues, it has become widely recognized that SC dysfunction could be at the base of the age-related overall deterioration in tissue and organ functionality (López-Otín et al., 2013; Schultz and Sinclair, 2016). In this regard, one of the main points of current research to improve a healthy lifespan focuses on enhancing the regenerative and repair capacity of adult tissue SCs.

It is unclear why SCs age. Although particular features differ among SCs in different organs, all SCs appear capable of self-renewing through asymmetric division and to move in and out of a tightly regulated quiescent state that likely preserves them from replication-related DNA damage (Schultz and Sinclair, 2016). In contrast to other somatic cells, telomerase expression is maintained in tissue-specific SCs after birth, but nonetheless telomeres exhibit attrition in SC populations at advanced ages (Martínez and Blasco, 2017). Furthermore, increased levels of p16^*INK*4*a*^ or p21^*CIP*1^ are found in some SCs in the elderly (Schultz and Sinclair, 2016; Herranz and Gil, 2018). Independently of whether SCs undergo cell senescence or not with age, they could sense the consequences of the gradual accumulation of senescent cells in tissues. In fact, certain SASP factors are able to induce senescence in adjacent cells in a paracrine manner (Coppé et al., 2010; Muñoz-Espín and Serrano, 2014). The secretome composition varies according to the cell origin, tissue location, or even the senescence inducer. Moreover, while some of these secreted factors could be restricted to particular tissues, others circulate in the systemic milieu (Coppé et al., 2010).

The adult mammalian brain contains neurogenic niches harboring neural SCs (NSCs) that sustain continual production of neurons for specific circuits throughout life. Despite their persistence, the rate of adult neurogenesis decays over time for reasons that are only partially understood. Although both intrinsic and extrinsic factors may likely underlie the reduction in adult neurogenesis observed with age, NSCs in the elderly can be re-activated to some extent, for example by physical exercise (Lugert et al., 2010), suggesting that cell-extrinsic factors play an important role in the age-related neurogenic decline. Here, we will propose the possibility that circulating and/or regional factors secreted by senescent endothelial cells (ECs) could account, at least in part, for the loss of NSC potential. To do so, we will first review the disturbances that occur in brain SC neurogenic activity with age. Then, we will focus on the vascular regulation of SC behavior in the young and aged neurogenic niches, paying special attention to vascular senescence and the effect of specific changes in the systemic composition of the blood. Finally, we will explore new perspectives regarding therapies for brain rejuvenation by targeting vascular aging.

## Adult Mammalian Neurogenesis and Its Decline With Age

**FIGURE 2:**
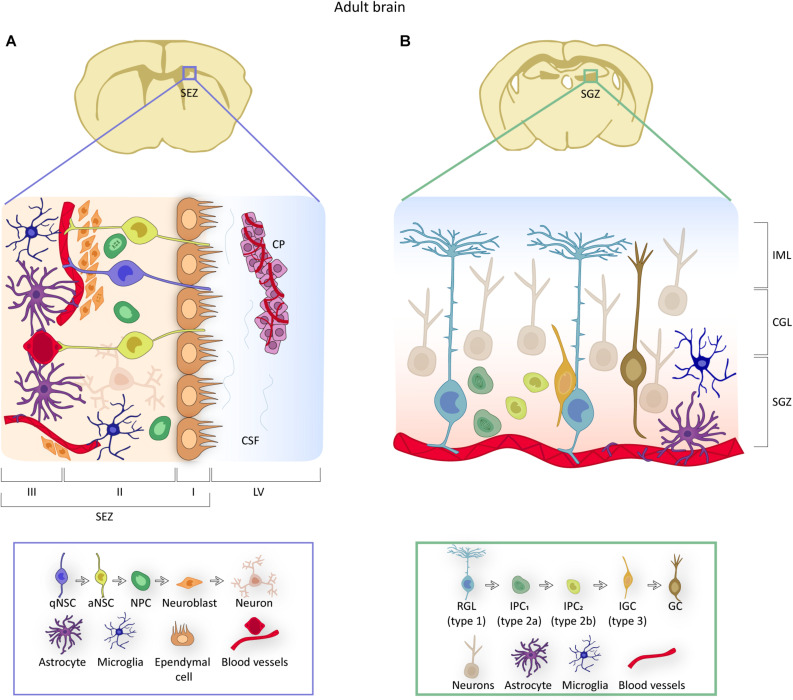
NSCs in adult brain neurogenic niches. **(A)** Representation of the location and organization of the SEZ neurogenic niche. NSCs contact the ependymal cell layer apically and the blood vessels through a basal process. Due to their specific position, NSCs receive signals from the choroid plexus (CP) through their access to the CSF (domain I), from other cell components of the niche (domain II) and from the vasculature (domain III). The box below identifies the components of the niche and provides an overview of the NSC lineage within the SEZ, from quiescent (q) to activated (a) NSCs to neural progenitor cells (NPCs) to neuroblasts. **(B)** Illustration of the location and organization of the hippocampal neurogenic niche. In the SGZ, NSCs or RGLs contact blood vessels and give rise to intermediate progenitor cells (IPCs) that progress into type 3 neuroblasts or intermediate granule cells (IGCs), to finally differentiate into granule cells (GC) that are located in the granule cell layer (GLC) and the inner molecular layer (IML). Mature granule cells constitute the neuronal circuitry of the DG. The box below includes an overview of the RGL lineage.

Self-renewing life-long NSCs reside in two distinct regions of the adult mammalian brain. In the subependymal zone (SEZ; also called ventricular-subventricular zone, or V-SVZ), NSCs give rise to neurons destined to integrate into olfactory bulb (OB) circuits whereas those in the subgranular zone (SGZ) of the dentate gyrus (DG) generate granule cells for the DG itself (Silva-Vargas et al., 2013; Ruddy and Morshead, 2018). The SEZ is by large the most active neurogenic niche in the murine brain, producing thousands of new neurons *per* day (Figure 2A; Obernier and Alvarez-Buylla, 2019). Subependymal NSCs derive from fetal radial glial cells from which they inherit an elongated morphology and biochemical features of the astrocytic lineage, such as expression of glial fibrillary acidic protein (GFAP), brain lipid binding protein (BLBP), or glutamate and aspartate transporter (GLAST, also known as SLC1A3) (Silva-Vargas et al., 2013). These NSCs (also known as B1 cells) are adjacent to the ependymal cell layer that lines the lateral ventricles (LV). They exhibit a thin apical cytoplasmic process that intercalates among ependymocytes ending in a primary cilium immersed into the ventricle cerebrospinal fluid (CSF) and a basal extension that moves away from the ventricle to end up at the basal lamina of the capillaries that irrigate the SEZ (Figure 2A; Silva-Vargas et al., 2013; Obernier and Alvarez-Buylla, 2019). Adult subependymal NSCs are specified during mid fetal development and most remain quiescent until adulthood (Fuentealba et al., 2015; Furutachi et al., 2015). When activated, NSCs divide symmetrically. While a 20% fraction of these divisions are self-renewing, most are consuming and produce transit-amplifying neural progenitor cells (NPCs, also known as C cells) which proliferate 3-4 times before they commit to differentiate, mostly into neuroblasts (Figure 2A; Ponti et al., 2013; Obernier and Alvarez-Buylla, 2019). Newly generated neuroblasts themselves can divide once or twice and then move anteriorly forming the rostral migratory stream toward the distant OB, where they terminally differentiate into interneurons involved in odor discrimination (Katsimpardi and Lledo, 2018). NPCs can also progress into oligodendroblasts destined to the corpus callosum to a lesser extent (Silva-Vargas et al., 2013).

Many subependymal NSCs were regarded as quiescent as they could retain traceable nucleosides and survived anti-mitotic treatments (Doetsch et al., 1999; Maslov et al., 2004; Mich et al., 2014). However, nucleoside-based cumulative labeling indicated that a fraction of these cells is fast-dividing, suggesting cycling heterogeneity (Ponti et al., 2013). Labeling with antibodies to different surface antigens and/or expression of a GFAP-driven GFP reporter, together with binding of fluorescent epidermal growth factor (EGF), indeed allowed the isolation of subsets of EGFR^*low/*–^ quiescent (q) and EGFR^+^ activated (a) subependymal NSCs by fluorescence-activated cell sorting (FACS) (Pastrana et al., 2009; Beckervordersandforth et al., 2010; Codega et al., 2014; Mich et al., 2014). In parallel, new technologies of deep RNA sequencing at the single-cell level of GLAST^+^ cells indicated the existence of an ordered progression from a ‘dormant’ deeply quiescent state (q), through one that is ‘primed’ (p) for activation, to an active (a) state (Llorens-Bobadilla et al., 2015). This information has led to optimization of the isolation procedure to obtain populations of aNSCs, pNSCs and qNSCs from the SEZ for functional analysis and to the realization that only aNSCs, with high levels of EGFR, and pNSCs, with a quiescent molecular signature and very low levels of EGFR, grow and generate neurospheres *in vitro* (Belenguer et al., 2020).

Onset of neurogenesis in the SGZ takes place when quiescent radial glia-like cells (RGL or type-1 cells) generate proliferative intermediate progenitors (IPCs, also known as type-2 cells or D cells). These cells are more differentiated and less frequently dividing than NPCs in the SEZ, but ultimately generate neuroblasts that migrate very short distances into the overlying DG granule cell layer and mature into granule interneurons (Figure 2B; Ruddy and Morshead, 2018). Hippocampal NSCs also exhibit molecular heterogeneity with dynamic expression profiles delineating a progression from quiescence to neuronal maturation (Shin et al., 2015; Artegiani et al., 2017).

It is widely accepted that advancing age correlates with a progressive reduction in the neurogenic output of both SGZ and SEZ niches (Kuhn et al., 1996; Luo et al., 2006; Ahlenius et al., 2009; Ben Abdallah et al., 2010; Lugert et al., 2010; Encinas et al., 2011; Conover and Shook, 2011; Shook et al., 2012). Traditional analyses had shown a decrease in the number of proliferative cells with increasing age, suggesting that the reduced level of neurogenesis in the elderly was primarily due to reduced numbers of NSCs (Maslov et al., 2004; Molofsky et al., 2006; Luo et al., 2006; Ahlenius et al., 2009). Those remaining in the aged SEZ were described as primarily quiescent as they appeared less likely to undergo cell division than NSCs in the young adult brain. Cycling NSCs, however, appeared more proliferative than their young counterparts, showing increased rates of cell cycle re-entry and transient expansion before lineage progression (Stoll et al., 2011), suggesting potentially complex modifications of cycle dynamics and gene expression (Daynac et al., 2016; Ziebell et al., 2018; Shi et al., 2018).

Intrinsic mechanisms underlying the neurogenic decline are still not fully understood. Reduced levels of specific growth factors and their signaling receptors are found in the aged SEZ (Tropepe et al., 1997; Enwere et al., 2004) whereas the expression of p16^*INK*4*a*^ and p19^*ARF*^ tumor suppressors increases with age in dividing cells, reducing cell proliferation (Molofsky et al., 2006; Bruggeman et al., 2005; Nishino et al., 2008). In this line, primary culture of neurospheres from a mouse model prone to accelerated senescence (SAMP8) has revealed that p19 and p53 are prematurely up-regulated and that both cooperate in cell cycle detention resulting in precocious senescence in the cultures (Soriano-Cantón et al., 2015). Some neurosphere cells exhibit telomere shortening and increased p53 levels after a number of passages. Indeed, telomerase activity decreases with age both in expression and activity leading to a decrease in NSC proliferation and p53 activation (Ferrón et al., 2004, 2009). However, although an increase in p21 following activation of p53 appears to be a trigger of senescence in many cells (Herranz and Gil, 2018), NSCs exhibit p53-independent high levels of p21 that are essential for the maintenance of their stem properties (Marqués-Torrejón et al., 2013; Porlan et al., 2013). Deficient asymmetric segregation of damaged proteins during NSC division and impaired proteostasis also correlate with NSC aging (Moore et al., 2015; Leeman et al., 2018). Recent deep-sequencing analyses of cardinal stem cell features over time has indicated an increase in the proportion of NSCs with a quiescence-related molecular signature in the elderly (Apostolopoulou et al., 2017; Ziebell et al., 2018; Kalamakis et al., 2019). Besides, Kalamakis and collegues have shown that this change is accompanied by an increase in the resistance of old quiescent NSCs to enter activation. Once the resistance to activation is overcomed, young and old NSCs show similar levels of proliferation and differentiation potential (Kalamakis et al., 2019). This correlates with observations of reactivation by external stimulation, i.e., exercise (Lugert et al., 2010). The data together suggests that NSCs are less likely to become activated in the context of an aged environment. Therefore, studying the extrinsic signaling involved in this phenomenon could be key to understand the age-related decline in neurogenesis. In this review we specifically consider those signals released by vascular cells and/or those that can reach neurogeneic niches through circulation.

## The Neurogenic Vascular Microenvironment and Its Transformation Over Time

Adult NSCs co-exist with multiple cellular components that condition their state and behavior through diverse mechanisms, including soluble factors and cell-cell interactions (Silva-Vargas et al., 2013). A unique feature of the SEZ is its proximity to the LV, which allows NSCs to receive signals from the CSF, through receptors at the membrane of their primary cilium, and from neighboring ependymal cells through cell adhesive interactions (Lehtinen et al., 2013; Silva-Vargas et al., 2016; Morante-Redolat and Porlan, 2019). In the distal or basal domain, at a distance from the ventricle lumen, the close contact with blood vessels enables NSCs to access both local factors (derived from vascular cells) and distant (blood-borne) molecules that permeate the SEZ parenchyma (Tavazoie et al., 2008; Shen et al., 2008; Mirzadeh et al., 2008; Rafii et al., 2016; Smith et al., 2018). Although NSCs in the SGZ are deeper in the brain parenchyma without direct access to the CSF compartment, they have a unique radial process that extends through the granular layer and an intimate contact with blood vessels beneath the DG (Palmer et al., 2000; Vicidomini et al., 2020).

**FIGURE 3:**
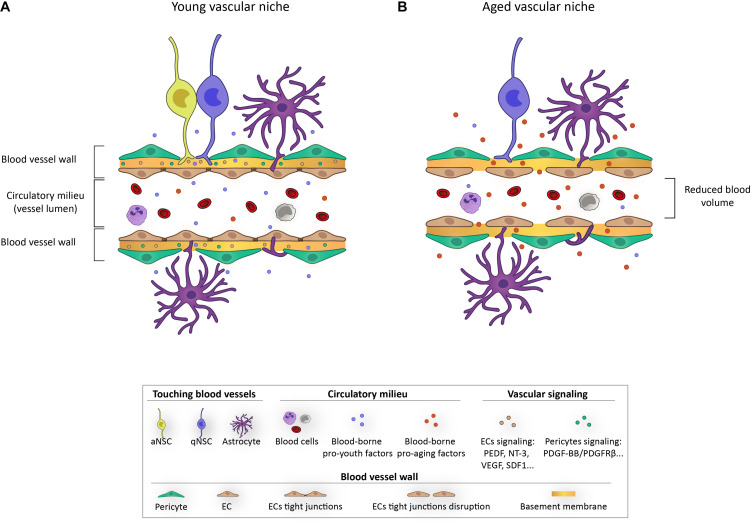
Schematic representation depicting the impact of aging on the vascular niche.

Blood vessels that irrigate the central nervous system have a specialized structure that is essential for homeostasis maintenance: the blood-brain-barrier (BBB). Differently from other organs, ECs are firmly sealed by tight junctions at the luminal face, lack fenestrations, and exhibit very low transcellular transport. This flat epithelium is enveloped by mural cells with contractile properties, such as vascular smooth muscle cells in large vessels and pericytes in capillaries, and astrocytic end-feet that also participate in flux regulation and vascular-to-neuronal communication (Obermeier et al., 2013). A basement membrane rich in different extracellular matrix (ECM) components ensheath all these cellular elements (Morante-Redolat and Porlan, 2019). This unique multicellular assembly constitutes an interface between the blood and the neural parenchyma and tightly regulates the trespass of blood-borne molecules and immune cells to the brain (Obermeier et al., 2013). The vasculature of neurogenic niches has, however, distinguishing features and a unique architecture. Unlike the tortuous morphology of blood vessels in non-neurogenic regions, an extensive planar vascular plexus runs parallel to the SEZ and NSCs and NPCs localize adjacent to this vascular plexus and directly contact blood vessels at specialized sites that lack astrocytic end-feet and pericyte coverage (Figure 3A; Shen et al., 2008; Tavazoie et al., 2008). Remarkably, the specialized BBB makes this neurogenic region more permeable to the trespass of systemic signals from the blood that, therefore, will be accessible to NSCs (Tavazoie et al., 2008). The DG vascular plexus also differs in its structure from the rest of the hippocampus and the pattern appears to be regulated by NPCs (Pombero et al., 2018). Activity of pre-existing DG neurons increases blood flow and permeability to growth factors that promote survival of newly born neurons (Shen et al., 2019).

The vascular niche in germinal centers provides a very rich source of regulatory signals that include locally secreted and systemically delivered factors, as well as cell-cell and cell-ECM interactions. Diffusible signals from ECs, known as angiocrine factors, appear important for homing, self-renewal, proliferation, and differentiation of NSCs and their progeny (Figure 3A). Among these, pigmented epithelium-derived factor (PEDF) was the first EC-derived soluble factor described to be involved in NSC regulation in the SEZ, causing an increase in self-renewal by enhancing Notch signaling (Ramírez-Castillejo et al., 2006; Andreu-Agulló et al., 2009). Thereafter, β-cellulin (Gómez-Gaviro et al., 2012), placental growth factor 2 (PIGF-2) (Crouch et al., 2015), or the chemokine stromal-derived factor SDF1 (Kokovay et al., 2010; Zhu et al., 2019) released by the vascular endothelium have been described to promote NSC proliferation. Contrary, other soluble factors, as for example neurotrophin-3, appear involved in quiescence maintenance (Delgado et al., 2014). NSC-vascular signaling mediated by cell adhesion has been considerably less studied (Kazanis et al., 2010; Morante-Redolat and Porlan, 2019). A pioneer study determined that NSC binding to ECM components of blood vessels through α6β1 integrins was essential for quiescence (Shen et al., 2008). Since then, a few more studies have associated quiescence with physical contact with vasculature, including laminin-binding through integrins (Kazanis et al., 2010) or cell-to-cell signaling involving Ephrin-Ephs and Notch-Notch ligands (Ottone et al., 2014).

With aging, the neurogenic niches are remodeled both structurally and functionally (Luo et al., 2006; Conover and Shook, 2011). Aging drives LV stenosis and progressive loss of ependymal cells, with a notable increase in the number of astrocytes that are interposed into the ependymal layer (Luo et al., 2008; Capilla-Gonzalez et al., 2014). Moreover, the remaining ependymal cells display altered morphology and protein expression and exhibit numerous lipid droplets, which reflect an altered cellular metabolism that has been associated with an increase in pro-inflammatory cytokine secretion (Capilla-Gonzalez et al., 2014; Ogrodnik et al., 2019). Astrocytes and microglia have been shown to suffer functional loss with age and even manifest a senescent profile *in vitro* in response to different stressors and *in vivo* (Conover and Shook, 2011; Capilla-Gonzalez et al., 2014; Cohen and Torres, 2019; Ogrodnik et al., 2021). Importantly, one of the most affected components with aging is the vasculature. Different vascular elements become dysfunctional during natural aging and alterations include increased permeability, ECM remodeling, altered BBB transport, endothelial and pericyte degeneration, and astrocyte malfunctioning. Hence, barrier integrity appears compromised in aged individuals, leading to abnormal trespassing of blood-borne proteins and impairment of blood flow regulation (Obermeier et al., 2013; Segarra et al., 2021). The described changes during aging, not only alter signaling interactions between vascular elements and NSCs, but also limit the supply of nutrients and oxygen and expose the niche to pro-aging circulating factors (Figure 3B; Silva-Vargas et al., 2013; Apple and Kokovay, 2017; Smith et al., 2018).

## Endothelial Cell Senescence and Its Associated Effects

Different types of glial cells and neurons have been shown to exhibit cell senescence traits in the aged rodent brain and to contribute to cognitive and neurogenic impairment as well as neurodegeneration (Jurk et al., 2012; Bussian et al., 2018; Ogrodnik et al., 2019; Zhang et al., 2019; Ogrodnik et al., 2021). However, functional studies of age-related EC senescence are still missing. The senescence associated β-galactosidase (SA-β-gal) histochemical reaction commonly used to discriminate senescent cells, which have increased levels of this lysosomal enzyme (Debacq-Chainiaux et al., 2009), usually correlates with other senescence-related traits in cultured ECs (i.e., primary human umbilical venous ECs, hUVECs) under pro-senescence pharmacological conditions (Kurz et al., 2000; Romero et al., 2019). However, a systematic description of whether SA-β-gal staining can be used to reliably monitor and measure EC senescence *in vivo* is still lacking, with very limited examples reported (Minamino et al., 2002). Nonetheless, with increasing age, ECs exhibit a number of phenotypic changes, including enlarged size, reduced proliferation, increased secretory activity, signs of DNA damage as well as higher levels of p21^*CIP*1^ and p16^*INK*4*a*^ or p53 (Zhan et al., 2010; Bhayadia et al., 2016; Regina et al., 2016; Jia et al., 2019; Grosse et al., 2020; Mühleder et al., 2020). In humans, the age-related increase in ECs with these characteristics correlates with vascular dysfunction (Table 1) (see Katsuumi et al., 2018 for a review).

ECs in aged mice exhibit transcriptomes that differ from those of young ECs (Leeman et al., 2018; Kalamakis et al., 2019; Kiss et al., 2020). Changes in secreted soluble factors by ECs can lead to altered signaling in the vascular neurogenic niche (Apple and Kokovay, 2017). Many of the upregulated genes are closely related to the molecular profile of senescent cells and to biological programs of response to cell damage, release of pro-inflammatory factors and generation reactive oxygen species (ROS), among others (Zhang et al., 2019; Kalamakis et al., 2019; Kiss et al., 2020). In particular, bulk RNA-Seq analysis of subependymal NSCs, microglia and ECs obtained from 2 and 19 month old mice revealed that ECs exhibit the most dramatic changes in transcripts related to inflammatory cytokines that promote NSC quiescence in the elderly (Kalamakis et al., 2019). Furthermore, senescent hUVECs secrete increased numbers of small extracellular vesicles (Riquelme et al., 2020) and EVs can induce paracrine senescence in healthy cells (Fafián-Labora et al., 2020).

From a metabolic perspective, p53 activation following DNA damage in senescent ECs affects glucose homeostasis and reduces the activity of nitric oxide synthase (eNOS) (Yokoyama et al., 2014), leading to a reduction in the expression of proliferator-activated receptor-gamma coactivator-1a (PGC-1a), with the subsequent impairment in mitochondrial biogenesis (Yokoyama et al., 2014). Senescence-associated mitochondrial impairment (Chapman et al., 2019) affects ECs considerably as these cells heavily rely on mitochondria to maintain normal function of ATP-dependent transporters for the regulation of vascular exchange. Their energy shift toward a more glycolytic profile is likely to sustain the high metabolic demands of the SASP (Sabbatinelli et al., 2019). Furthermore, NO controls vasodilatation and, therefore, the reduced amounts resulting from the reduced expression and/or activity of eNOS (Van Der Loo et al., 2000; Matsushita et al., 2001) could be causing a hypoperfusion of brain cells, lowering the biodisponibility of nutrients and oxygen at brain (Graves and Baker, 2020). In this sense, aging also entails a decrease in the expression of GLUT1 in ECs, leading to a reduced transport of glucose across the BBB that interferes with the metabolism of surrounding cells (Lee et al., 2018).

Given the close proximity of NSCs to the blood vessels, the metabolic rewiring associated to vascular senescence could have important consequences for tissue renewal. Indeed, the metabolic changes promoted by EC senescence could influence the metabolic transition from glycolysis to oxidative phosphorylation that occurs when qNSCs become activated (Llorens-Bobadilla et al., 2015), acting as a contributing factor to the diminished propensity of NSCs to activate as age advances. Furthermore, EC senescence induces a systemic metabolic dysfunction through actions in the white adipose tissue, increasing insulin resistance in the organism as part of the metabolic syndrome (Barinda et al., 2020). Paracrine induction of a senescence-associated phenotype is, therefore, accompanied by a reduced expression of insulin receptor substrate (IRS) and insulin resistance. This systemic effect could be detrimental for NSC maintenance, as the insulin signaling pathway is key to NSC proliferation (Chirivella et al., 2017). Moreover, high levels of circulating glucose, as a consequence of metabolic syndrome, downregulate eNOS activity, as well as the activity of telomerase (Hayashi et al., 2006), leading to the spread of senescence.

Dysfunctional mitochondria produce high levels of reactive oxygen species (ROS) which act as spreaders of senescence in a paracrine way. ROS promote DNA damage (Srinivas et al., 2019) and also act as scavengers for NO, generating peroxinitrite, that inhibits mitochondrial superoxide dismutase (SOD2) thus increasing O_2_^–^ (Van Der Loo et al., 2000). The excess of O_2_^–^ can damage mitochondrial complexes generating a positive loop for enhanced mitochondrial ROS generation (Maranzana et al., 2013). In addition, SOD1 (cytosolic) or SOD2 deficiency has been also associated with the senescent phenotype (Treiber et al., 2012; Zhang et al., 2017), as well as the increased activity of NADPH oxidase (NOX), specially NOX1, both factors contributing to the high levels of ROS (Salazar, 2018). The increase in oxidative stress that occurs in aging is also partially related to pericyte alterations. These cells increase the expression of inducible NOS (iNOS), leading to increased NO that may derive in increased peroxynitrite when reacting with O_2_^–^ anions (Van Der Loo et al., 2000). However, determining the exact role of ROS in senescence, as inductors or byproducts, is difficult. Overall, EC senescence displays an altered metabolism and promotes pro-oxidant and pro-inflammatory stimuli which correlates with a dysfunctional vascular profile (Regina et al., 2016; Katsuumi et al., 2018). Therefore, it could contribute to the deterioration of the neurogenic activity in the aged niches.

## The Aging Systemic Milieu: Blood-Borne Effects on Neurogenesis

The composition of plasma varies with age and the idea of blood-borne factors as systemic inducers of age-associated features has strongly emerged from observations in heterochronic parabionts. In parabiosis, a young and an old mouse share their circulatory system following a surgical joining of their belly skin that results in a long-lasting anastomosis of capillaries (Villeda et al., 2011). Because effects in parabionts could be related to a prolonged sharing of blood circulation, the effects of a transient blood exchange through a jugular venous catheter connection have also been investigated (Rebo et al., 2016). These types of studies have revealed that, similarly to SCs in other organs, old blood reduces neurogenesis in young mice while young blood rejuvenates the neurogenic potential of elderly mice (Villeda et al., 2011; Katsimpardi et al., 2014; Smith et al., 2015; Rebo et al., 2016; Smith et al., 2018; Yousef et al., 2019). Intringuely, short-term exposure of old mice to young blood does not recapitulate the brain rejuvenating effects of the heterochronic parabiosis, suggesting that old blood has a very potent anti-neurogenic function (Rebo et al., 2016). Studies using either heterochronic parabiosis or transient blood exchange have identified specific blood-borne factors that increase with age and can negatively influence adult neurogenesis. Reported pro-aging factors that impair adult neurogenesis include β2-microglobulin (B2M), transforming growth factor beta 1 (TGFβ1), chemokine (C-C motif) ligand 11 and 2 (CCL11 and CCL2), interleukin 6 (IL-6), and tumor necrosis factor α (TNF-α) (Villeda et al., 2011; Smith et al., 2015; Rebo et al., 2016; Smith et al., 2018). In contrast, certain factors detected in young blood are notably reduced in circulation during aging. Some of the reported pro-youthful factors with neurogenesis enhancing effects are growth differentiation factor 11 (GDF11) and the hormone oxytocin (Leuner et al., 2012; Katsimpardi et al., 2014). Other hormones appear to improve neuroblast survival (Kase et al., 2020) and young blood restores levels of IGF-I, growth hormone, Wnt3, TGF -β and GDF11 in old mice (Schultz and Sinclair, 2016).

Considering the protein nature of some of these factors and the existence of the BBB, it is surprising that they can have an impact on the neurogenic niches. Direct actions on NSCs and/or surrounding niche cells (i.e., microglia, astrocytes, or pericytes) couls indicate unforeseen BBB trespassing (Erickson et al., 2014). However, the potential mechanisms involved in these effects remain largely unexplored, waiting for the characterization of receptors and signaling mechanisms involved. Indirect mechanisms have also been considered, as vascular function and blood flow can affect neurogenesis. For example, blood vessel angiograms and volumetric analyses in the SEZ have revealed the benefits of young blood for the restoration of vascular architecture in aged mice. GDF11 pro-youth effects in SEZ-dependent neurogenesis are accompanied by vessel remodeling, blood flow improvement, and enhanced angiogenesis (Katsimpardi et al., 2014). More research will be needed to characterize the mechanistic underpinnings of these effects.

The chemoquine CCL11/Eotaxin-1 was the first reported pro-aging plasma factor with detrimental effects on DG neurogenesis. CCL11 levels in blood increase significantly with aging and both systemic and intracerebral administration of CCL11 to young mice result in decreased hippocampal neurogenesis, an effect that can be rescued with neutralizing anti-CCL11 antibodies (Villeda et al., 2011). Reduced neurogenesis is also observed when B2M is systemically injected in young mice whereas aged B2M-deficient mice exhibit increased learning and memory skills relative to controls, as well as a notably increased neurogenic activity (Smith et al., 2015). However, it is still unknown if B2M crosses the BBB, and recent studies suggest that B2M increases only in certain tissues, likely in response to other pro-aging stimuli (Yousef et al., 2015). Although the specific mechanism by which B2M mediates neurogenesis dysfunction is not yet clear, the inflammation-related increase in B2M suggests that a process of cell senescence could mediate its expression in the DG (Smith et al., 2015). Furthermore, several studies have identified B2M as a marker of senescence and even some nanodevices have been reported to detect senescent cells based on the recognition of B2M (Althubiti et al., 2014; Paez-Ribes et al., 2019).

TGF-β1 is a pleiotropic cytokine which is up-regulated with age both in the blood and in the hippocampus. Apart from its multi-faced role in the immune response, it is also one of the SASP cytokines and can induce cell senescence (Rea et al., 2018). Its depletion enhances DG neurogenesis in aged mice (Yousef et al., 2015). Furthermore, as it mediates inflammation, its attenuation leads to reduced B2M levels (Yousef et al., 2015; Rebo et al., 2016), revealing a possible link between both pro-aging factors. The age-related burden of senescent cells in concert with the growing levels of TGF-β1 promotes inflammation and oxidative stress that have been related to serious pathological effects within the brain (Tominaga and Suzuki, 2019), including vascular disturbances and the disruption of the BBB (Rebo et al., 2016; Rea et al., 2018). The SASP component CCL2, has also been proposed as a potential negative regulator of adult neurogenesis (Lee et al., 2013). IL-6 and TNF-α, also identified as components of the SASP are associated with premature vascular senescence, disruption of endothelial tight junctions, and vascular hypertrophy and dysfunction (Schrader et al., 2007; Kojima et al., 2013; Khan et al., 2017; Didion, 2017; Jia et al., 2019; Basisty et al., 2020). During aging there is an increase in the blood circulation of IL-6 and TNF-α, which also corresponds to a higher expression of these factors in the choroid plexus (CP) (Smith et al., 2018). Likewise, heterochronic reconstitution experiments have also recently indicated that old hematopoietic cells reduce hippocampal neurogenesis through increased levels of cyclophilin A, a SASP factor (Smith et al., 2020). Together, these changes may account for the accumulation of pro-aging SASP factors in the old blood, which are also likely to promote senescence induction at the vascular level, ultimately contributing to the deterioration of adult neurogenesis.

The CSF constitutes an important nexus of signaling between the blood stream and the SEZ (Lehtinen et al., 2013). This enriched fluid is mainly produced by the CP, which consist of a highly vascularized structure floating in the brain ventricles (Lehtinen et al., 2013) making it likely sensitive to changes in soluble factors present in the blood. Indeed, the CP acquires an inflammatory transcriptional profile over time (Silva-Vargas et al., 2016) and elevated levels of different cytokines, such as IL-6 and CCL11, have been reported in the CSF when young animals are exposed to an old bloodstream (Smith et al., 2018). Therefore, changes in the blood vessels during aging may ultimately be responsible for altering the secretory activity of the CP and the composition of the CSF, which disrupts its normal function, affecting NSC behavior in the SEZ.

Studies that establish a direct relation between old-blood effects and cell senescence are scarce. However, some recent investigations using heterochronic parabiosis have analyzed the effects of the young blood on aged kidneys and visceral adipose tissue (VAT), in which cell senescence, accompanied by decreased autophagy and elevated inflammation levels, becomes prominent with increasing age. The results demonstrated that a young blood environment significantly reduces kidney and VAT cell senescence, reduces pro-inflammatory cytokines and SASP precursors, lowers the levels of p16^*INK*4*a*^ and p21^*CIP*1^ and restores some indicators of autophagy (Huang et al., 2018; Ghosh et al., 2019). These studies suggest that circulating factors in the young plasma disrupt senescence phenotypes and reverse the inflammatory status in tissues. Therefore, preventing age-related accumulation of these factors could potentially boost neurogenesis in the aging brain.

## Brain Rejuvenation: Science or Science Fiction?

Although the underlying mechanisms involved in the loss of NSC neurogenic potential with age have not yet been clarified, cell senescence arises as an important driver of age-related alterations and chronic inflammation in brain (Apple and Kokovay, 2017). Aging drives the accumulation of senescent cells at the systemic level affecting multiple tissues and leading to pathological consequences in both tissue function and architecture (Van Deursen, 2014; Yousefzadeh et al., 2020). A weakened immune phagocytic response and anti-apoptotic pathways (SCAP) selectively activated by senescent cells are also likely to significantly contribute to the increasing burden of senescent cells with age (Zhu et al., 2015; Ovadya et al., 2018). Senescence in the aged brain affects different niche components, especially vasculature. Therefore, cell senescence has been introduced herein as a potential candidate for triggering the deleterious effects of aging observed in the neurogenic niches. In this regard, vascular cell senolysis could represent a promising target to rejuvenate neurogenic regions and to promote brain health, either alone or combined with interventions that target metabolism, such as caloric restriction or type 2 diabetes-treating drug metformin (Cutler and Kokovay, 2020; Zhu et al., 2020).

The most promising senolytic drugs mediate the selective elimination of these cells through the inhibition of their pro-survival mechanisms (Zhu et al., 2016). Because the SCAP network can vary in different senescent cells, senolytic drugs that target a specific SCAP only remove a subset of cells (Zhu et al., 2016; Hickson et al., 2019). For example, Navitoclax (ABT-263) inhibits the anti-apoptotic proteins BCL-2, BCL-W and BCL-X1, upregulated in senescent cells, and induces apoptosis on hUVECs and fibroblasts of the IMR90 cell line *in vitro* (Zhu et al., 2016; Yosef et al., 2016). In addition, its oral administration in elderly mice has been shown to promote the elimination of senescent SCs in the bone marrow and muscle leading to the rejuvenation of these tissues (Chang et al., 2016). One of the most widely used strategies to clear senescent cells has been the combination of two senolytic drugs, dasatinib (D) and quercetin (Q). D inhibits tyrosine kinases related to antiapoptotic pathways. Instead, Q is a flavonoid that targets BCL-2 family members, HIF-1α and specific nodes in PI3-kinase and p21 that are involved in SCAPs and leads to the removal especially of senescent ECs (Zhu et al., 2016; Hickson et al., 2019). The mixed use of D + Q orally decreases senescent cell incidence *in vivo* with beneficial effects on mouse tissue renewal and health span (Xu et al., 2018; Ogrodnik et al., 2019; Ogrodnik et al., 2021). Furthermore, clinical trials are being performed using D + Q to effectively eliminate senescent cells in humans with different pathologies (e.g., Hickson et al., 2019). Interestingly, these drugs are able to cross the BBB and have been described to eliminate senescent periventricular cells in the SEZ of obese mice, improving neurogenesis (Ogrodnik et al., 2019). Importantly, D + Q exerts beneficial effects at the vascular level in elderly mice, improving carotid artery relaxation, which leads to an enhancement of the vasomotor function due to the re-activation of the signaling pathways involved in hemodynamic responses (Roos et al., 2016). This promising result suggests that senolysis may lead to the reversal of age-related vessel remodeling with consequences on adult neurogenesis. This could be essential for the development of new therapies aimed at recovering the neurogenic function of NSCs and extending brain health longevity. Therefore, although there is still a lot to be done in the field, rejuvenating brain tissue through neuronal regeneration, is increasingly far from science fiction. However, further studies should be done to transform it to a scientific reality.

## Conclusion and Future Perspectives

Aging is a negative regulator of adult neurogenesis. The proportion of deeply quiescent NSCs increases with age and the capacity of self-renewal and regeneration is reduced, resulting in a loss of the neurogenic function in the aged neurogenic niches (Conover and Shook, 2011; Kalamakis et al., 2019). NSC activity is tightly regulated by multiple intrinsic and extrinsic signals especially from blood vessels. Senescent cells progressively accumulate in tissues with increasing age and, through the SASP, contribute to chronic inflammation and lead to profound tissue deterioration. In this sense, vascular cells acquire a senescent phenotype with age at the brain level, which have been reported to actively contribute to neuroinflammation (Kalamakis et al., 2019; Kiss et al., 2020). Regarding the neurogenic niches, this could account for the age-related loss of neurogenesis, since senescence impairs the role of ECs. Importantly, the studies reviewed herein add to the link between senescence and aging, but also support our view of endothelial senescence as a potential anti-neurogenic target.

Endothelial cells senescence also contributes to the disruption of the BBB integrity (Yamazaki et al., 2016; Jia et al., 2019). The age-related alteration of the BBB leads to an abnormal exposure of the NSCs to blood circulating factors that may mediate anti-neurogenic effects and parabiosis studies highlight the role of the systemic milieu in neurogenesis modulation (Smith et al., 2018). Heterochronic parabiosis studies have demonstrated that young blood reduces the inflammatory profile of tissues in older mice leading to the reduction of senescent markers (Ghosh et al., 2019; Huang et al., 2018) and highlight the significance of the deterioration of blood vessels and/or milieu to the age-related neurogenesis decline (Smith et al., 2018).

In addition, it is likely that blood-derived factors may trigger pro-aging effects in the blood vessels themselves, which in turn also affects neurogenesis. Nevertheless, the mechanisms by which extrinsic factors, either from the vascular niche or the systemic environment, disrupt NSC activity during aging still needs to be completely defined (Villeda et al., 2011; Smith et al., 2015; Rebo et al., 2016). Indeed, whether aged blood and the factors contained therein are able to induce senescence in young mice remains yet to be established. In this sense, the identification of endogenous factors that can promote the spread of senescence could be interesting targets to delay the age-related accumulation of senescent cells and their adverse consequences. For instance, the chemokine CCL11 has been described to increase in blood plasma and in the CSF with age, and has been shown to impair adult neurogenesis within the SGZ (Villeda et al., 2011). It is possible that certain senescent cells secrete CCL11 (locally or/and systemically), or perhaps some SASP factors stimulate the expression of this chemokine with age in blood.

Regarding the design of therapies aimed at restoring neurogenesis, the inhibition of pro-aging or senescence-associated factors can be a strategy to consider. However, it could be more efficient to target the senescent cells themselves in order to have more promising and lasting results. Although, this line of research has yet to be fully attained, this approach could be useful to stablish a relationship between old blood components and senescence in a vascular context, as well as to stablish a therapeutic pathway of action. In the recent years, senolysis has emerged as a potentially promising intervention for tissue rejuvenation. The removal of senescent cells helps to reduce inflammation and promotes tissue renewal (Xu et al., 2018; Ogrodnik et al., 2019). Therefore, the proper detection and elimination of senescent cells become reasonable targets for establishing a therapeutic approach to prevent brain decline. However, the specific effects of senolytic drugs on the deteriorated neurogenic function of elderly mice deserve more investigation. Furthermore, the long-term use of these drugs may be limited by their off-target and adverse side effects (Ghosh et al., 2019; Ogrodnik et al., 2019). Indeed, recent analyses in a mouse line genetically modified to selectively expressed the diphtheria toxin receptor in p16-expressing senescent cells found that senescent ECs in the liver selectively ablated upon treatment with diphtheria toxin were not replaced by new ones, creating important endothelial disruptions (Grosse et al., 2020). Therefore, many more studies are needed to understand potentially distinct responses of ECs in different organs.

In this sense, it may also be interesting to look for therapies that not only remove senescent cells, but also promote tissue regeneration. In line with this idea, combined therapies including senolytic treatment and the administration of pro-youthful factors, could represent a promising strategy. For instance, the GDF11 factor stimulates SEZ vascular rejuvenation, which may contribute to the restoration of the proper extrinsic regulation that ECs exert on NSC behavior, and to improve neurogenesis (Katsimpardi et al., 2014). Accordingly, this review proposes endothelial senescence as a driver of the decreased neurogenic potential observed with age, and as a target for the design of therapies aimed at restoring adult neurogenesis. Ultimately, the objective is to repair damaged neural circuits and reduce the impact of age-associated neurodegenerative disorders, or at least delay their development, by rejuvenating brain tissue and extending brain health.

## Author Contributions

All authors listed have made a substantial, direct, and intellectual contribution to the work and approved it for publication.

## Conflict of Interest

The authors declare that the research was conducted in the absence of any commercial or financial relationships that could be construed as a potential conflict of interest.
